# Commentary: Mindfulness training for reducing anger, anxiety, and depression in fibromyalgia patients

**DOI:** 10.3389/fpsyg.2016.00740

**Published:** 2016-05-19

**Authors:** Lorys Castelli, Valentina Tesio

**Affiliations:** Department of Psychology, University of TurinTurin, Italy

**Keywords:** mindfulness, fibromyalgia, psychotherapy, anger, depression

In their recent study, Amutio et al. (2015) aimed to “verify whether the application of a mindfulness-based training program was effective in modifying anger, anxiety, and depression levels” in women with fibromyalgia and “whether the changes achieved were maintained over time.”

Fibromyalgia (FM) is a chronic syndrome characterized by widespread musculoskeletal pain, non-restorative sleep, fatigue, but also cognitive impairment (Tesio et al., 2015), mood disorder (Wolfe et al., 2010; Consoli et al., 2012) and emotional dysregulation disorders, such as alexithymia (Di Tella and Castelli, 2013; Martínez et al., 2015). Since FM has a severe negative impact on the patients' quality of life (Castelli et al., 2012; Segura-Jiménez et al., 2015) and causes severe disability in daily activities (Consoli et al., 2012; Schaefer et al., 2015), studies on the effectiveness of treatments, in particular psychotherapeutic ones (Castelnuovo, 2010), are crucial. The best approach seems to be the integration between pharmacological and non-pharmacological treatments while engaging patients as active participants in the process (Clauw, 2014). Different psychotherapeutic approaches have been used and, currently, Mindfulness-Based Therapies (MBTs) are one of the most promising techniques.

Mindfulness can be described as bringing one's complete attention and awareness to the current experiences, including aversive ones, in a non-judgmental and accepting way (Kabat-Zinn, 1990). It is a valuable approach for learning new non-reactive models of responding to the emotional discomfort, through a process that enables thoughts and feelings to be experienced as subjective (instead of necessary) and transient (instead of permanent). Whereas, Mindfulness-Based Cognitive Therapy (MBCT) was originally developed as a psychological treatment for relapse prevention in recurrent depression (Segal et al., 2002), Mindfulness-Based Stress Reduction (MBSR) intervention was developed specifically to help people to manage pain and the stress associated with long-term conditions (Kabat-Zinn, 2003). By interrupting maladaptive automatic responses, mindfulness may prevent negative affective cascades and facilitate a greater awareness of available positive affective sources. This in turn may modulate subjective experience of pain and disability, enhancing general features of coping with distress and disability in everyday life (Ludwig and Kabat-Zinn, 2008).

A systematic meta-analysis of six MBSR trials for FM revealed short-term improvement of QoL and pain after MBSR, when compared to both usual care and active control interventions (Lauche et al., 2013). In their review on MBTs efficacy in the treatment of somatization disorders, Lakhan and Schofield (2013) concluded that, in FM patients, symptom severity improved after MBTs. More recently, another systematic review analyzed the effectiveness of MBTs in managing FM symptoms (Henke and Chur-Hansen, 2014). Even though the wide variation in reported outcome measures did not allow a direct comparison of results, the synthesis resulted in an overall positive evidence for the efficacy of MBTs. In particular, patients in the mindfulness groups reported an improvement in pain, sleep, or psychological distress (Henke and Chur-Hansen, 2014). These reviews underline the potential for MBTs to enhance self-management of FM symptoms, but they also recommend large-scale randomized controlled clinical trials in order to draw more definitive conclusion, especially for long-term effects.

Despite the relatively small sample size, the study of Amutio et al. (2015) has the methodological value of assessing the long-term effect of a mindfulness-based training program on a group of women with FM. What is more, this study could be considered promising in terms of achieved results, in particular with regards to anger reduction. The mindfulness intervention program implemented by Amutio et al. (2015) (2-h session for seven consecutive weeks) included the following characterizing elements: exercises originally used by Kabat-Zinn, mindfulness-related strategies used in acceptance and commitment therapy, explanations and discussions of the metaphors and exercises applied, tales taken from Zen philosophy and Vipassana meditation. The training aimed to “the re-education of conditioned and automatic ways of reacting” when faced with thoughts, emotions, and sensations. In this way, patients “learn how to break the habit of letting their thoughts, emotions, and sensations control them,” learning to let thoughts flow and observing how they come and go without trying to change them (Amutio et al., 2015). Amutio and colleagues focused, in particular, on the evaluation of the efficacy of the mindfulness-based intervention in modifying anger, anxiety and depression levels. Previous studies had, in fact, showed the the ability to express emotion in FM patients was associated with a lower impact of fibromyalgia (Geenen et al., 2012) and that chronic angry emotional reactions, leading to pervasive interpersonal disruption and chronic sympathetic activation, are often maladaptive (Sayar et al., 2004).

The authors used a quasi-experimental design, comparing an experimental group (*N* = 20) and a waiting-list control group (*N* = 19). Furthermore, the authors performed a follow-up evaluation after 3 months (Amutio et al., 2015). After the mindfulness-based training, FM patients showed significant improvement in state anger, internal anger expression, internal control of anger, state anxiety and depression (Amutio et al., 2015). Furthermore, many of the benefits were maintained after the 3-months follow-up period, in particular those concerning depression, state anxiety, internal expression and control of anger. The improvements obtained in internal control and expression of anger are particularly important since the use of adaptive coping strategies to face emotional expression and control has been associated with lower impact of FM (Geenen et al., 2012). By means of the modification of the maladaptive strategies, MBTs could result not only in a better emotion regulation, but also in a global improvement of the FM syndrome.

Treatment guidelines for the management of FM symptoms emphasize the importance of patient education and self-care strategies, and all recommend integrating non-pharmacologic with pharmacologic treatment (Menzies, 2016). Amutio and colleagues highlighted the effectiveness of mindfulness not only for the treatment of anxiety and depression in FM syndrome, but also the feasibility of mindfulness-based intervention on anger. In addition the authors demonstrated that this beneficial effect keeps unchanged at long-term follow up. This last result seems to confirm that mindfulness training, altering the way mental processes and contents are experienced, does not limit itself to directly reduce pain intensity but, going further, it establishes a new mindful, accepting and long-lasting pain coping style.

## Author contributions

Both the authors have written the manuscript after a critically read of the target article; in addition both the authors gave the final approval of the manuscript submitted for publication.

## Funding

LC was supported by University of Turin grants (“Ricerca scientifica finanziata dall'Università” Linea Giovani; http://www.unito.it). VT was supported by CRT Foundation project “Componenti psicologiche e psicosomatiche nella sindrome fibromialgica”. The funders had no role in decision to publish or preparation of the manuscript.

### Conflict of interest statement

The authors declare that the research was conducted in the absence of any commercial or financial relationships that could be construed as a potential conflict of interest. The reviewer, EG, and handling Editor declared their shared affiliation, and the handling Editor states that the process nevertheless met the standards of a fair and objective review.
